# Diplomas in Radiography

**Published:** 1921-08-13

**Authors:** 


*To the Editor of* The Hospital.

### Diplomas in Radiography.

*To the Editor of* The Hospital.

Sir,—I was interested to see in your issue of July 30 "Sparkgap's" letter referring to the Society of Radiographers. Some months ago I wrote to the Secretary of the Society asking for a syllabus of the work required by the candidates for examination. This I received the last week in July, when the examination was evidently over, as the names of successful candidates appeared in your paper on July 23. I agree with "Sparkgap" that anyone having ten or more years' experience in radiography, or holding a certificate of a recognised training-school, such as Guy's, should automatically be accepted in a newly-formed Society. I happen to know that many large hospitals in England and abroad employ radiographers trained at Guy's Hospital, and holding their certificate. Surely this fact speaks for itself as to the efficiency of the training?

I remain, yours faithfully,

